# The Monoclonal Antitoxin Antibodies (Actoxumab–Bezlotoxumab) Treatment Facilitates Normalization of the Gut Microbiota of Mice with *Clostridium difficile* Infection

**DOI:** 10.3389/fcimb.2016.00119

**Published:** 2016-10-04

**Authors:** Mária Džunková, Giuseppe D'Auria, Hua Xu, Jun Huang, Yinghua Duan, Andrés Moya, Ciarán P. Kelly, Xinhua Chen

**Affiliations:** ^1^Área de Genómica y Salud, Fundación para el Fomento de la Investigación Sanitaria y Biomédica de la Comunidad ValencianaValencia, Spain; ^2^Instituto Cavanilles de Biodiversidad y Biología Evolutiva, Universitat de ValènciaValencia, Spain; ^3^Centro de Investigación Biomédica en Red en Epidemoioloía y Salud PúblicaMadrid, Spain; ^4^Division of Gastroenterology, Beth Israel Deaconess Medical Center, Harvard Medical SchoolBoston, MA, USA

**Keywords:** *Clostridium difficile* infection, antibiotics, Bayesian networks, *C. difficile* toxin antibody, 16S rDNA amplicon sequencing, MK-3415A, actoxumab and bezlotoxumab, immune therapy

## Abstract

Antibiotics have significant and long-lasting impacts on the intestinal microbiota and consequently reduce colonization resistance against *Clostridium difficile* infection (CDI). Standard therapy using antibiotics is associated with a high rate of disease recurrence, highlighting the need for novel treatment strategies that target toxins, the major virulence factors, rather than the organism itself. Human monoclonal antibodies MK-3415A (actoxumab–bezlotoxumab) to *C. difficile* toxin A and toxin B, as an emerging non-antibiotic approach, significantly reduced the recurrence of CDI in animal models and human clinical trials. Although the main mechanism of protection is through direct neutralization of the toxins, the impact of MK-3415A on gut microbiota and its restoration has not been examined. Using a CDI murine model, we compared the bacterial diversity of the gut microbiome of mice under different treatments including MK-3415A, vancomycin, or vancomycin combined with MK-3415A, sampled longitudinally. Here, we showed that *C. difficile* infection resulted in the prevalence of *Enterobacter* species. Sixty percent of mice in the vehicle group died after 2 days and their microbiome was almost exclusively formed by *Enterobacter*. MK-3415A treatment resulted in lower *Enterobacter* levels and restoration of *Blautia, Akkermansia*, and *Lactobacillus* which were the core components of the original microbiota. Vancomycin treatment led to significantly lower survival rate than the combo treatment of MK-3415A and vancomycin. Vancomycin treatment decreased bacterial diversity with predominant *Enterobacter* and *Akkermansia*, while *Staphylococcus* expanded after vancomycin treatment was terminated. In contrast, mice treated by vancomycin combined with MK-3415A also experienced decreased bacterial diversity during vancomycin treatment. However, these animals were able to recover their initial *Blautia* and *Lactobacillus* proportions, even though episodes of *Staphylococcus* overgrowth were detected by the end of the experiments. In conclusion, MK-3415A (actoxumab–bezlotoxumab) treatment facilitates normalization of the gut microbiota in CDI mice. It remains to be examined whether or not the prevention of recurrent CDI by the antitoxin antibodies observed in clinical trials occurs through modulation of microbiota.

## Introduction

*Clostridium difficile* infection (CDI) has become the leading recognized cause of nosocomial infectious diarrhea in the developed world and with increased incidence and mortality rates in recent years (Evans and Safdar, 2015). Antibiotics have significant and long-lasting impacts on the intestinal microbiota and consequently reduce colonization resistance against CDI. Although antibiotic usage is a major risk factor for acquiring CDI, the standard care in clinics still remains the use of antibiotics (metronidazole or vancomycin). Significant portion of CDI patients treated with metronidazole or vancomycin will suffer a recurrence after treatment is discontinued; many of these will have multiple recurrences (Kelly and LaMont, 2008; O'Horo et al., 2014). Given the increasing CDI incidence and severity as well as high rate of disease recurrence after standard therapy, it is clear that current approaches to disease prevention and treatment are inadequate and that new non-antibiotic approaches are sorely needed (Lowy et al., 2010; Evans and Johnson, 2015; Lee et al., 2016).

It is already known that the normal fecal microbiome provides a barrier against *C. difficile*, and that antibiotic therapy weakens or disrupts this barrier allowing colonization. The idea that restoration of the gut microbiota is necessary to prevent recurrence is supported by an ~90% success rate of fecal microbiota transplantation (FMT; Seekatz et al., 2015), and significant recovery of diversity and community membership has been observed following FMT treatment for recurrent CDI (Hamilton et al., 2013; Song et al., 2013; Seekatz et al., 2014). Specific microbiota-derived characteristics associate with disease severity and recurrence have been demonstrated (Seekatz et al., 2016), although the precise composition of such a protective barrier is not yet known (Gerding and Johnson, 2010).

Therefore, emerging CDI therapies are focused on limiting further perturbation of the colonic microbiota and/or restoring the microbiota to its pre-morbid state, reducing colonization of gut by pathogenic *C. difficile* and bolstering the host immune response against its toxins (Kociolek and Gerding, 2016). As an emerging non-antibiotic therapy, human monoclonal antibodies MK-3415A (actoxumab–bezlotoxumab) target *C. difficile* toxins, the major virulence factors of *C. difficile*, rather than the organism itself. Actoxumab (formerly known as MK-3415, GS-CDA1, and MDX-066) and bezlotoxumab (formerly known as MK-6072, MBL-CDB1, and MDX-1388) target toxin A and toxin B, respectively. MK-3415A, the combination of actoxumab–bezlotoxumab, significantly reduced the recurrence of CDI in animal models (Hernandez et al., 2015; Yang et al., 2015; Zhang et al., 2015) and human clinical trials (Lowy et al., 2010), although in phase III trial co-administration with actoxumab did not provide additional benefit. Although the main known mechanism of protection is through direct neutralization of the toxins (Hernandez et al., 2015; Yang et al., 2015), the impact of MK-3415A on gut microbiota and its restoration has not been examined.

One potential mechanism for reduced recurrence with anti-toxin therapy is colonic toxin neutralization, thereby preventing harmful toxin effects and leading to a curative restoration of a healthy microbiome. Therefore, we hypothesize that MK-3415A exerts its protective effect against CDI recurrence by facilitating the restoration of the normal microbiota, in opposite to the negative effects of standard antibiotic treatments such as vancomycin. To test this hypothesis, we longitudinally monitored the dynamics of gut microbial profiles of mice that were infected with *C. difficile* and then subsequently treated with MK-3415A and/or vancomycin.

## Methods

### Mice experiments

**Figure 1:**
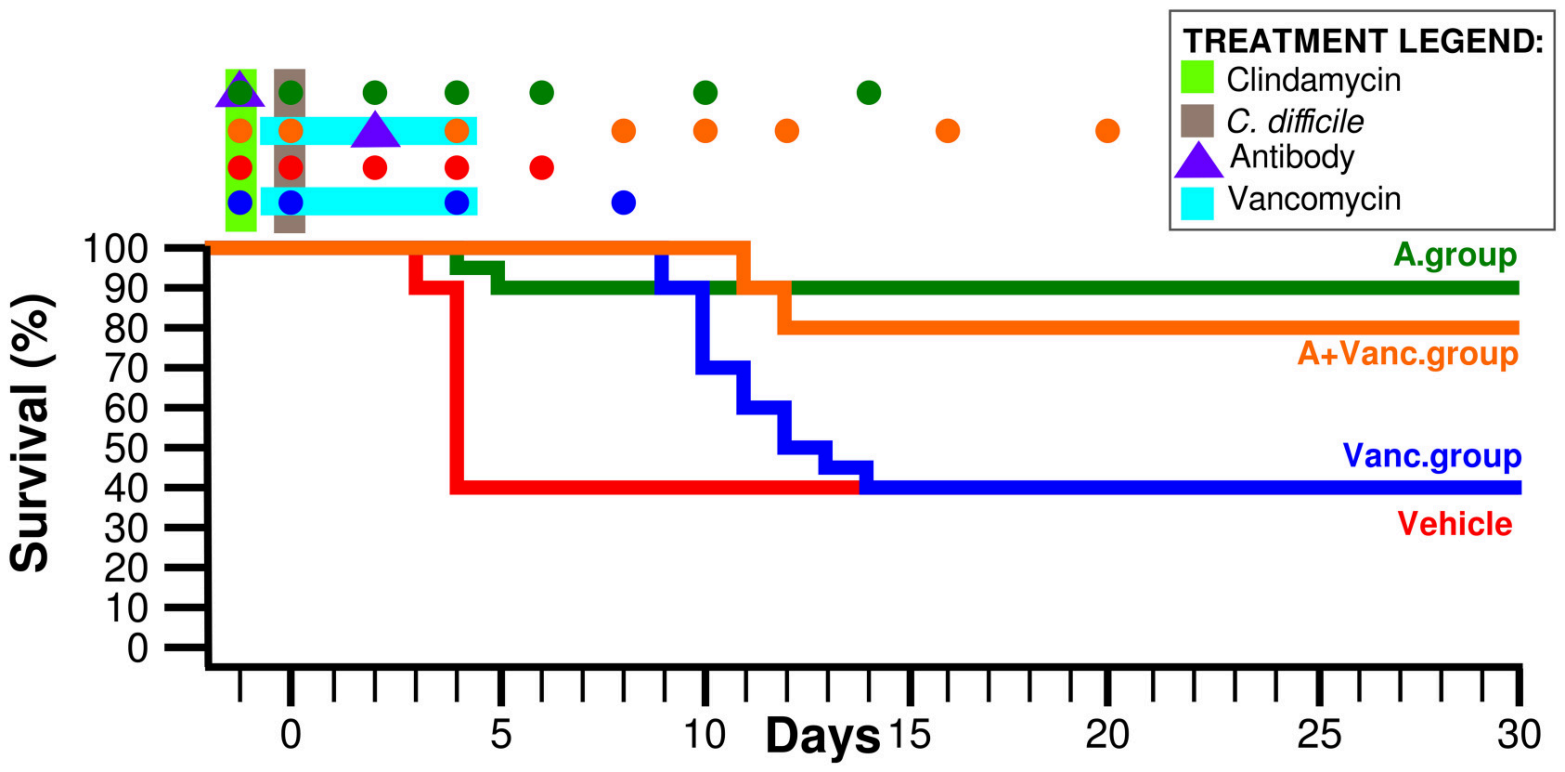
**Mice survival rate of the four experimental groups**. Time-points in which the fecal samples were collected are marked by colored dots above the graph. The days of antibiotic treatment and antibody administration are also marked above the graph as explained in the legend.

The murine CDI model was done as previously described (Chen et al., 2008). MK-3415A or vehicle was given to clindamycin-exposed mice 1 day prior to *C. difficile* inoculation (day −1) at a sub-lethal dose. Mice that developed severe CDI were sacrificed and cecal contents were collected. The antibody group (*n* = 10) has been treated with MK-3415A (250 μg actoxumab and bezlotoxumab each, Merck Inc.) 1 day before *C. difficile* infection. The vancomycin group has been treated with oral vancomycin (50 mg/kg) during the days 0–4. The last group contained mice (*n* = 10) treated with the antibody (on day 2) and with vancomycin (days 0–4) at the same doses as above. There was also a control mice group treated by a vehicle (*n* = 10). Individual mouse stool samples were collected on days −1, 0, 2, 4, 6, 8, 10, 14, 16, and 20 (depending on survival or disease status) as shown in the Figure 1. In total, 183 fecal samples were processed. Survival rate and weight as development signs of CDI were recorded from the day 0 to the day 28. All animal experiments were performed in accordance with the guidelines of the IACUC committee of Beth Israel Deaconess Medical Center.

### DNA extraction

The mouse stool samples (about 100 μl) were re-suspended in 1 ml of phosphate-buffered saline (PBS), vortexed rigorously and let to pellet on the bench for about 30 min. The upper part was transferred to fresh 1.5 ml tubes and centrifuged at 13,000 g for 5 min. The bacterial pellet was washed twice with PBS and finally resuspended in 1 ml of PBS. An aliquot (220 μl) of the superior part was mixed with 350 μl of lysozyme (at conc. 10 mg/ml) and incubated 30 min at 37°C. Then 30 μl of sodium dodecyl sulfate (conc. 10%) and 1.6 μl of proteinase K (conc. 50 mg/ml) was added and incubated for 60 min at 50°C. Afterwards, 100 μl 5M NaCl and 80 μl cetyl trimethylammonium bromide (10% of CTAB in NaCl) was added, the tubes were vortexed rigorously and incubated for 15 min at 65°C. DNA purification was performed by addition of 700 μl of phenol followed by shaking for 2 min and centrifugation at 13,000 g for 3 min. The upper part was transferred to a new tube and mixed with 700 μl of chloroform and again shaken for 2 min and centrifuged. The upper part was mixed with 220 μl of 5 M NH_4_-acetate and 1 μl of glycogen. Afterwards, 500 μl of isopropanol was added, the tubes were mixed by inverting up and down and incubated at −80°C for 2 h. Afterwards, the tubes were centrifuged at 13,000 g for 45 min at 4°C. The supernatant was discarded and the pellet was washed by addition of 500 μl 70% ethanol and centrifugation for 10 min. The supernatant was again discarded and the pellet was let to dry out at room temperature and finally resuspended in 100 μl of water.

### 16S rRNA gene amplification and sequencing

The regions V3 and V4 of the 16S rDNA gene were amplified according to Klindworth et al. (2013). The PCR mix contained: 31.75 μl water, 4 μl dNTPs 2.5 mM each, 0.25 μl ExTaq polymerase 5 U/μl (Takara, Ref. RR001A), 5 μl ExTaq buffer 10x, 2 μl of 10 mM 16S rDNA forward primer (5′-TCG TCG GCA GCG TCA GAT GTG TAT AAG AGA CAG CCT ACG GGN GGC WGC AG-3′), 2 μl of 10 mM 16S rDNA reverse primer (5′-GTC TCG TGG GCT CGG AGA TGT GTA TAA GAG ACA GGA CTA CHV GGG TAT CTA ATC C-3′), and 5 μl DNA. PCR conditions: initialization step at 94°C for 2 min, 25 cycles of 94°C for 30 s, 54°C for 30 s, 72°C for 30 s, and the final elongation at 72°C for 10 min. The PCR reactions were purified by NucleoFast 96 PCR purification plates (Machinery-Nagel, Ref. 743100.10) and finally resuspended in 50 μl water. The purified PCR reactions were multiplexed using Nextera XT library preparation kit (Illumina, Ref. FC-131-1096) and prepared for sequencing on MiSeq Illumina platform according to the manufacturer's instructions (Illumina, Ref. MS-102-3001).

### Sequence processing

Sequence quality assessment was carried out by using PRINSEQ program (Schmieder and Edwards, 2011). Sequences of length <200 nt have not been considered; 5′ trimming was performed cutting out nucleotides with a mean quality <30 in 20 bp windows. Eventual chimeric 16S amplicons have been removed by USEARCH program (Edgar, 2010). The sequences have been deposited in European Nucleotide Archive database with study accession number PRJEB10920. Obtained sequences were clustered to form operational taxonomic units (OTUs) by CD-hit program at 0.95 similarity level (Li and Godzik, 2006) and they were associated with their taxonomic classification obtained by RDP_classifier (boostrap cut-off 0.8) program from Ribosomal Database Project (Cole et al., 2009). The bacterial OTUs that constituted for < 183 read (1 read/sample on average) have been joined into under-represented OTUs item.

### Bacterial diversity analysis

The Shannon diversity index was calculated using R package “vegan” (Oksanen et al., 2006). If all abundance is concentrated to one type, and the other types are very rare (even if there are many of them), Shannon index approaches zero. The changes in Shannon index along the time was analyzed by Wilcoxon test (*p* < 0.05) comparing the consecutive time-points of the experimental groups. In addition, the values of the day with the lowest bacterial diversity were compared with the last collected day of each experimental group.

### Canonical correspondence analysis

The canonical correspondence analysis (CCA) was used for the ordination of the 183 samples. Using the “envfit” function from “vegan” R package, the bacterial composition (OTUs with average proportion >0.02%) was tested for fitting on the time-points of the experimental groups categories. Mice were allocated in different experimental cages, thus the influence of cage on the microbiota was tested, too; for this test the samples were divided into corresponding experimental groups and time-points and a CCA using the “envfit” function has been carried out.

### Comparison of the experimental groups at their common time-points

The percentage proportions of OTUs (OTUs with average proportion >0.02%) have been compared by Wilcoxon test (significance level at *p* < 0.05) in the following experimental groups at their common time-points.

### Longitudinal analysis of OTUs proportion

A significant (*p* < 0.05) increase/decrease of proportion of each OTU among two consecutive time-points was analyzed for each individual mice by “edgeR” package (Robinson et al., 2010), which includes Benjamini-Hochberg correction (all considered OTUs were taken into this analysis). Only significant variations confirmed by at least 80% of mice were considered.

### Correlations among bacterial OTUs, body weight and experimental time

Bayesian networks constructed using R package “bnlearn” (Scutari, 2010) were used to find statistically significant correlations (Spearman correlation >0.3 and < −0.3) among the bacterial OTUs (average proportion >0.02%), experimental time-points (after day −1) and the mice body weight. Separate networks were constructed for each experimental mice group. The connecting arcs of such a network represent mutual associations (not causality). From a Bayesian network, a subset called Markov blanket can be extracted determining which bacterial taxa influences the selected nodes (Vazquez-Castellanos et al., 2015). In our case, the nodes corresponding to the body weight and time-points were dissected in order to describe the most important associations within each experimental group.

## Results

Ninety percent of mice in the MK-3415A antibody group (A.group) were symptomless during the whole experimental duration (Figure 1). The antibody + vancomycin group (A+Vanc.group) had the similar final survival rate of 80%. In contrast, in vancomycin group (Vanc.group) death occurred in 60% of mice; their life span (50% of mice surviving) was 10 days. The vehicle group had life span of only 4 days. The collected samples of the vehicle group were divided into two groups: the mice with immediate death (vehicle dead group—VehD.group) and the survived mice (vehicle alive group—VehA.group).

16S rDNA amplicons sequencing produced an average of 65,658.78 ± 40,677.07 sequences per sample. The dataset contained in total 12,316,896 sequences, which were clustered into 11,044 OTUs. The bar-plots representing the bacterial composition for all 183 samples are shown in the Figure S1.

**Figure 2:**
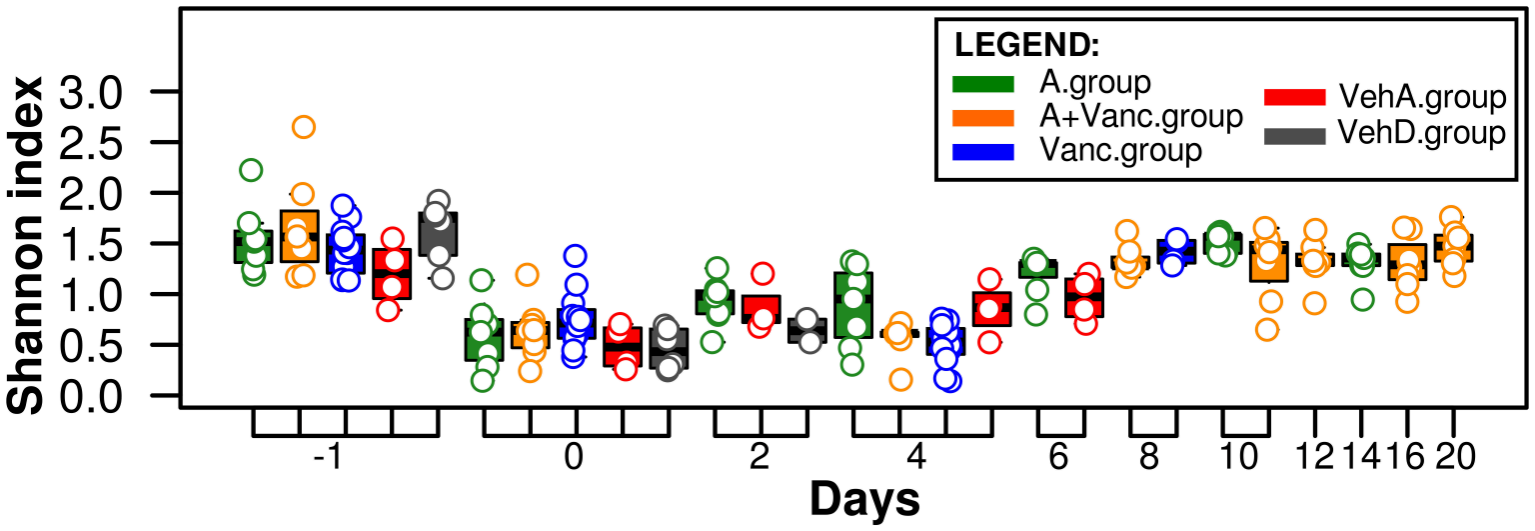
**Shannon microbial diversity index**. The values for each sample are visualized as colored dots according to their experimental groups (red, gray, blue, green, and orange). The microbiome of all experimental groups had the highest Shannon diversity index on day −1 and decreased significantly (*p* < 0.01) on day 0.

Day −1 refers to the day of clindamycin administration prior to *C. difficile* infection and was considered as a baseline of the mice microbiome after antibiotic mixture exposure. The microbiome of all experimental groups had the highest Shannon diversity index on day −1 (1.57 ± 0.38, Figure 2), which significantly decreased on day 0 (the day of *C. difficile* infection, 1 day after clindamycin administration). The diversity index decreased significantly on day 0 (*p* < 0.01 in all groups, Figure 2), with the bacterial diversity concentrating on a few of the most prevalent OTU. In the following days, the Shannon index differed between experimental groups. For example, in the vancomycin-treated experimental groups (Vanc.group and A+Vanc.group) low bacterial diversity was detected on day 4, but it returned to the initial values after the vancomycin treatment finalized (on the day 8).

**Figure 3:**
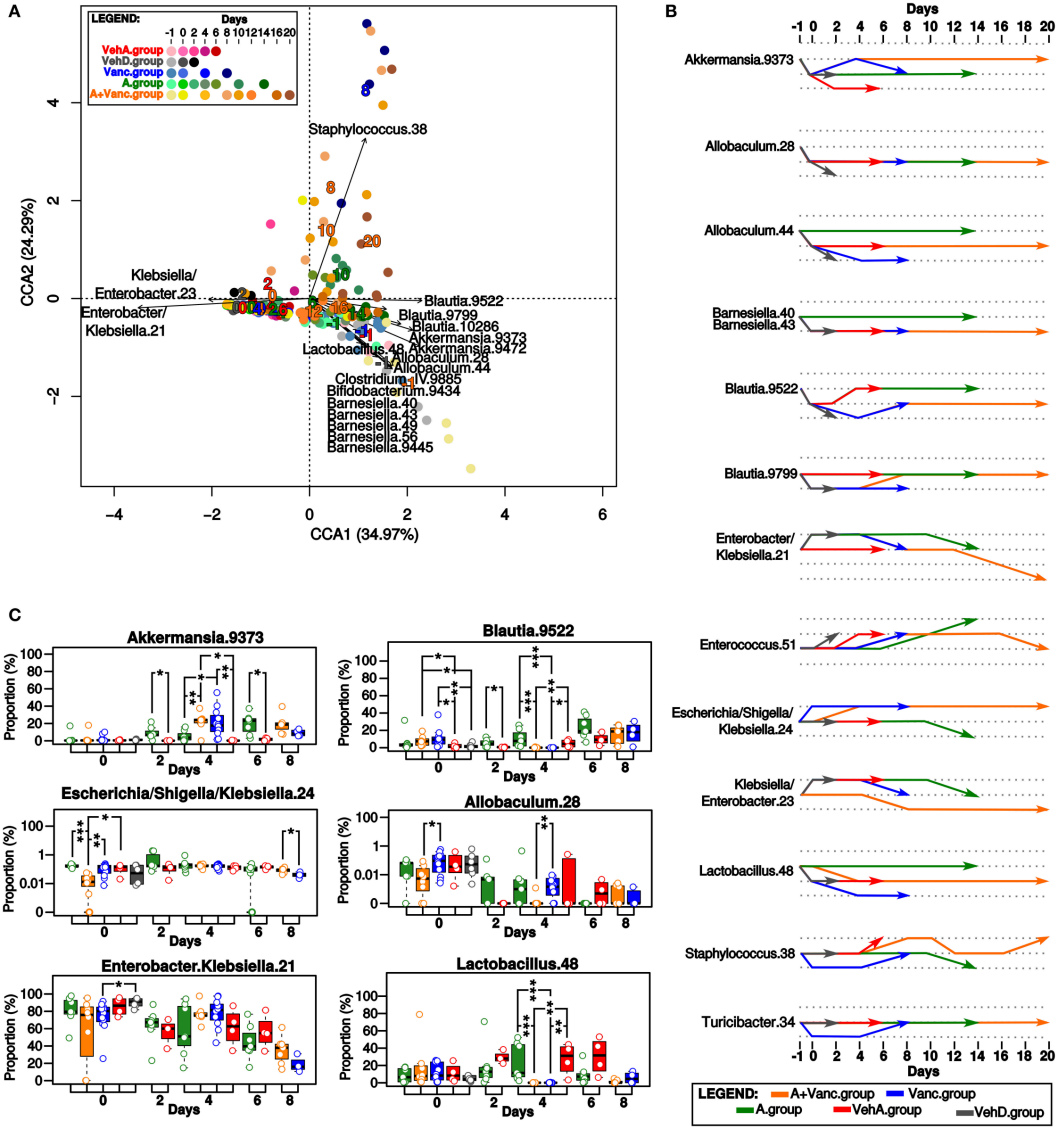
**Microbial composition of the experimental groups. (A)** The canonical correspondence analysis (CCA). The time-points of the experimental groups had significant influence on the microbial composition. Each dot represents the total microbial composition of one sample. The names of bacterial OTUs with the significant influence (*p* < 0.01) on the ordination of samples in this CCA plot are shown, it was mostly driven by tree bacterial groups (*Klebsiella/Enterobacter, Blautia/Akkermansia/Allobaculum/Lactobacillus/Barnesiella*, and *Staphylococcus*). The numbers in the CCA plot refer to the collected days, they are colored according to the experimental group colors (red, gray, blue, green, orange) and their coordinates in the plot are given by the direction of their influence on the ordination of the samples. **(B)** Longitudinal comparison of the proportion of bacterial OTUs in pairs of two consecutive time-points. The increasing/decreasing direction of the arrows mean that the proportion of that particular OTU had significant (*p* < 0.05) fold-change increase/decrease between the inquired consecutive time-points. The most important changes in bacterial proportions occurred on the days 0 and 4 corresponding to the treatment changes. **(C)** Comparison of the OTUs proportions in the common time-points of the experimental groups. Significant differences are marked by asterisks (^*^p < 0.05, ^**^p < 0.01, ^***^p < 0.001), box-plot colors refer to the experimental groups. The most important differences between groups were detected on day 0 and 4.

Afterwards, we analyzed the longitudinal changes in the proportions of the bacterial OTUs. Bacterial composition was significantly influenced by the type of treatment and the experimental time (*p* < 0.001, Figure 3A). In contrast, it was confirmed that grouping of mice by cages did not have significant influence on the microbiota composition (*p* > 0.05).

The most typical representative OTUs of the initial microbiome on day −1 in all experimental groups were *Akkermansia, Blautia, Lactobacillus, Allobaculum*, and *Barnesiella* OTUs (Figure 3). On day 0, drastic changes in bacterial composition occurred, these OTUs were replaced almost exclusively by *Enterobacter* and *Klebsiella* in all experimental groups. Proportions of other OTUs evolved differently depending on their experimental group. For example, *Lactobacillus* proportion significantly decreased (*p* < 0.05) on day 0 in all experimental groups except A.group (Figure 3B). A.group quickly recovered its initial proportions of *Blautia* and *Akkermansia*. In contrast, the high proportions of *Enterobacter*/*Klebsiella* (OTU 21) persisted in the VehA.group until the last experimental day and its microbiome did not recover to its initial composition.

The microbiome of the vancomycin-treated groups (Vanc.group and A+Vanc.group) was modulated differently from the antibiotic-free groups (Figure 3). On day 4, the microbiomes of vancomycin-treated groups were almost exclusively formed by *Enterobacter/Klebsiella* and *Akkermansia*. The amount of *Blautia* and *Lactobacillus* were significantly reduced, while the amount of *Akkermansia* was significantly increased in the vancomycin groups in comparison of those without vancomycin treatment (*p* < 0.05, Figure 3C). After termination of vancomycin treatment, the microbiome dominated by *Enterobacter/Klebsiella* and *Akkermansia* was replaced by one with higher proportions of *Blautia, Staphylococcus, Turicibacter*, and *Enterococcus*. A.group also contained low proportions of *Staphylococcus* on day 10. However, the proportion of *Staphylococcus* on days 8 and 10 in Vanc.group was significantly higher (*p* = 0.006).

Due to the short life span of the mice in the Vanc.group (the last sample collection was on day 8), it was not possible to compare microbiome patterns after vancomycin treatment with the microbiome of A+Vanc.group. The final experimental days of A+Vanc.group (days 10–20) had oscillating proportions of *Staphylococcus* (significantly decreased on days 12–16 and increased on day 20) and a decreasing trend of *Enterobacter/Klebsiella* resembling the original microbiome composition (Figure 3).

**Figure 4:**
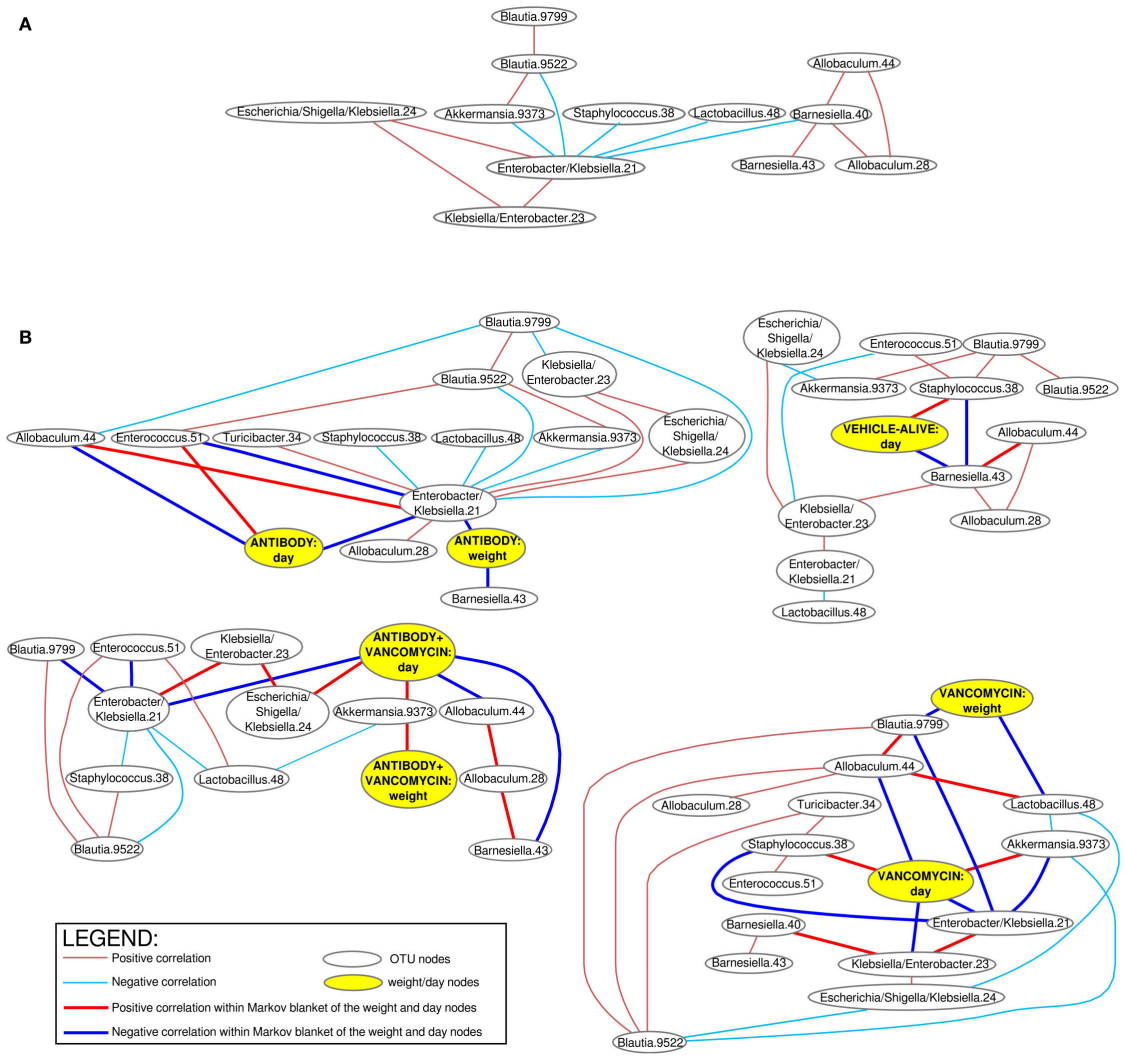
**Bayesian networks of correlations of the bacterial OTUs**. Positive (red arcs) and negative (blue arcs) correlations (Spearman correlation >0.3 and < −0.3) between bacterial OTUs visualized as a Bayesian network using R package “bnlearn.” The connecting arcs of the network represented mutual associations (not causality). Separate networks were constructed for all samples combined together **(A)** and also separated according to the experimental group **(B)**. Separate networks for each experimental group in the **(B)** contain also correlations with mice body weight and experimental time. The bold arcs in the **(B)** connect the bacterial OTUs with have the most important associations to the body weight and experimental time (so called dissection of Markov blankets). VehD.group was excluded from the analysis due to the low number of time-points.

The Bayesian networks based on microbial frequencies of all samples in all experimental groups (Figure 4A) showed that the *Enterobacter/Klebsiella* OTU 21 correlated negatively with *Akkermansia, Allobaculum, Barnesiella, Blautia, Lactobacillus, Staphylococcus*, and with the mice body weight.

When the Bayesian networks were analyzed individually for each experimental group (Figure 4B), we found that the body weight correlates positively with different OTUs, depending on the applied treatment. The body weight in the A.group correlated negatively with *Enterobacter/ Klebsiella*. In contrast, the body weight of the antibiotic treated groups (Vanc.group and A+Vanc.group) correlated negatively with *Blautia* and *Lactobacillus*. This was not expected, because *Enterobacter/ Klebsiella* and *Blautia*+*Lactobacillus* were antagonists. One likely explanation for this observation was that after the termination of vancomycin treatment the mice body weight of the vancomycin groups significantly increased (*p* < 0.01), but the proportions of *Blautia* and *Lactobacillus* decreased, as they were replaced by *Enterobacter/Klebsiella* and *Akkermansia* (day 4).

## Discussion

Human monoclonal antibodies MK-3415A to *C. difficile* toxin A and toxin B, as passive immune therapy, have been shown to significantly reduce the recurrence of CDI in human clinical trials (Lowy et al., 2010). Although the main mechanism of protection is through direct neutralization of the toxins (Yang et al., 2015), the impact of MK-3415A on gut microbiota and its restoration has not been examined. In this study we examined the impact of antitoxin antibodies on the gut microbiota and its restoration. Our results demonstrated that MK-3415A significantly influences the taxonomic composition of the gut microbiota. Clindamycin pre-exposure and *C. difficile* infection produced a decrease in microbial diversity caused by an overgrowth of *Enterobacter/Klebsiella* species. Microbiome of mice succumbed to *C. difficile* infection was characterized by high proportions of *Enterobacter/Klebsiella* species, while the mice treated by MK-3415A were able to reconstitute their original microbial composition formed mainly by *Lactobacillus, Akkermansia*, and *Blautia*. Vancomycin treatment resulted in decreased bacterial diversity and overgrowth of *Enterobacter/Klebsiella* and *Akkermansia* species. This was also observed in mice with combined treatment of vancomycin and MK-3415A, confirming the strong effect of vancomycin on microbiota. Vancomycin temporarily attenuated the disease, but with an increase of body weight observed right after the vancomycin treatment in the vancomycin and vancomycin + MK-3415A mice groups. However, mice treated by vancomycin died shortly after the treatment was terminated. In contrast, mice treated with vancomycin + MK-3415A were able to recover the proportion of *Blautia* and *Lactobacillus* with much higher survival rate.

*Akkermansia* was one of the most prevalent genera observed in the mice microbiota before clindamycin pre-treatment and *C. difficile* infection. *Akkermansia* is an intestinal mucin-degrading bacterium (Derrien et al., 2004). However, *Salmonella* mice infection models showed that the excessive mucin degradation by *Akkermansia* may contribute to inflammatory bowel diseases because mucin degradation facilitates the access of luminal antigens to the intestinal immune system (Ganesh et al., 2013). It is important to mention, that the original microbiota of our experimental mice before *C. difficile* infection contained also high amounts of *Lactobacillus* and *Blautia*. *Lactobacillus* species are often included in the probiotics or fecal transplantation mixtures due to their anti-inflammatory properties (Mohamadzadeh et al., 2011). *Blautia* has been associated with whole grain diet (commonly used for experimental mice feeding) and coincided with improvements in host physiological measures (Martinez et al., 2013). Vancomycin treatment in our study significantly decreased the proportions of *Lactobacillus* and *Blautia*, while the high amounts of *Akkermansia* persisted. In addition, the high proportion of *Akkermansia* after vancomycin treatment was accompanied by a high proportion of *Enterobacter/Klebsiella*. It might suggest that if high proportion of *Akkermansia* is accompanied with high proportions of *Lactobacillus* and *Blautia*, its inflammatory effect of *Akkermansia* might be attenuated. However, this is still very speculative and remains to be further examined.

The overgrowth of *Enterobacter/Klebsiella* after clindamycin pre-treatment is a reflection of its natural resistance to this antibiotic (Nyberg et al., 2007). Similar observations of increase of *Enterobacter* and decrease of *Barnesiella* after clindamycin pre-treatment and *C. difficile* inoculation in mice models has been reported (Buffie et al., 2012).

*Staphylococcus* is commonly sensitive to clindamycin (Levin et al., 2005) and therefore it was suppressed until the clindamycin effect completely disappeared. Interestingly, after the termination of vancomycin treatment, *Staphylococcus* and *Enterococcus* expanded to the levels higher than those at the beginning of the experiment or in the normal microbiota of the antibody group. *Staphylococcus* and *Enterococcus* can be found in human microbiota, but they are opportunistic pathogens which could take advantage of the decreased bacterial diversity due to vancomycin treatment to initialize their colonization (Hiramatsu, 2001; Kaslow and Shiver, 2011; Stecher et al., 2013). Indeed, it was well-documented that antibiotic treatment of *C. difficile* infections leads to the emergence of additional infections by bacteria resistant to these antibiotics (Willems et al., 2005).

Mice group treated with vancomycin and MK-3415A also experienced a reduction of bacterial diversity during the vancomycin treatment. However, these mice were able to recover their initial *Blautia* and *Lactobacillus* proportions. It is important to mention that the episodes of *Staphylococcus* overgrowth were detected during the final experimental days despite the MK-3415A administration. It might indicate that in opposite to MK-3415A alone, MK-3415A plus vancomycin may increase the risk of the infection by other opportunistic pathogens and *C. difficile* recurrence would persist due to the enormous ability of vancomycin to perturb the gut microbiome.

In conclusion, MK-3415A facilitates normalization of the gut microbiota in antibiotic-treated, *C. difficile*-challenged mice compared to the untreated animals. In contrast, vancomycin perturbs the gut microbiome, causes the overgrowth of potential opportunistic pathogens and reduces the amounts of anti-inflammatory bacterial species. Despite significant microbiome perturbations caused by vancomycin, antitoxin antibodies in combination with vancomycin still preserves its ability to normalize the gut microbiota and to improve disease outcome. This study may help explain the beneficial effect of MK-3415A observed in animal models and in clinical trials of CDI. The difference of impact on human or animal microbiota between the individual components (actoxumab vs. bezlotoxumab) is worthy of further investigation.

## Author contributions

XC is responsible for the study design, interpretation of data, drafting of the manuscript, and obtained funding. CK is responsible for the study design, interpretation of data, and obtained funding. MD, GD, and AM are responsible for acquisition, analysis and interpretation of data, drafting of the manuscript and obtained funding. HX, JH, and YD are responsible for acquisition of data.

### Conflict of interest statement

XC received research support from Merck Inc. through an investigator initiated study program on *C. difficile* infection. The other authors declare that the research was conducted in the absence of any commercial or financial relationships that could be construed as a potential conflict of interest.

## Supplementary material

Figure S1 **Bacterial proportions of all sequenced samples**. Bar-plots showing the percentage proportion of the 19 most prevalent bacterial OTUs. The OTUs summing <7% in total of all 183 samples together are joined into “Underrepresented_species.”
