# Supplementary figures and images for: The Monoclonal Antitoxin Antibodies (Actoxumab–Bezlotoxumab) Treatment Facilitates Normalization of the Gut Microbiota of Mice with Clostridium difficile Infection
Source: Front Cell Infect Microbiol. 2016 Oct 4;6:119. doi: 10.3389/fcimb.2016.00119 (PMC5048712; doi:10.3389/fcimb.2016.00119)

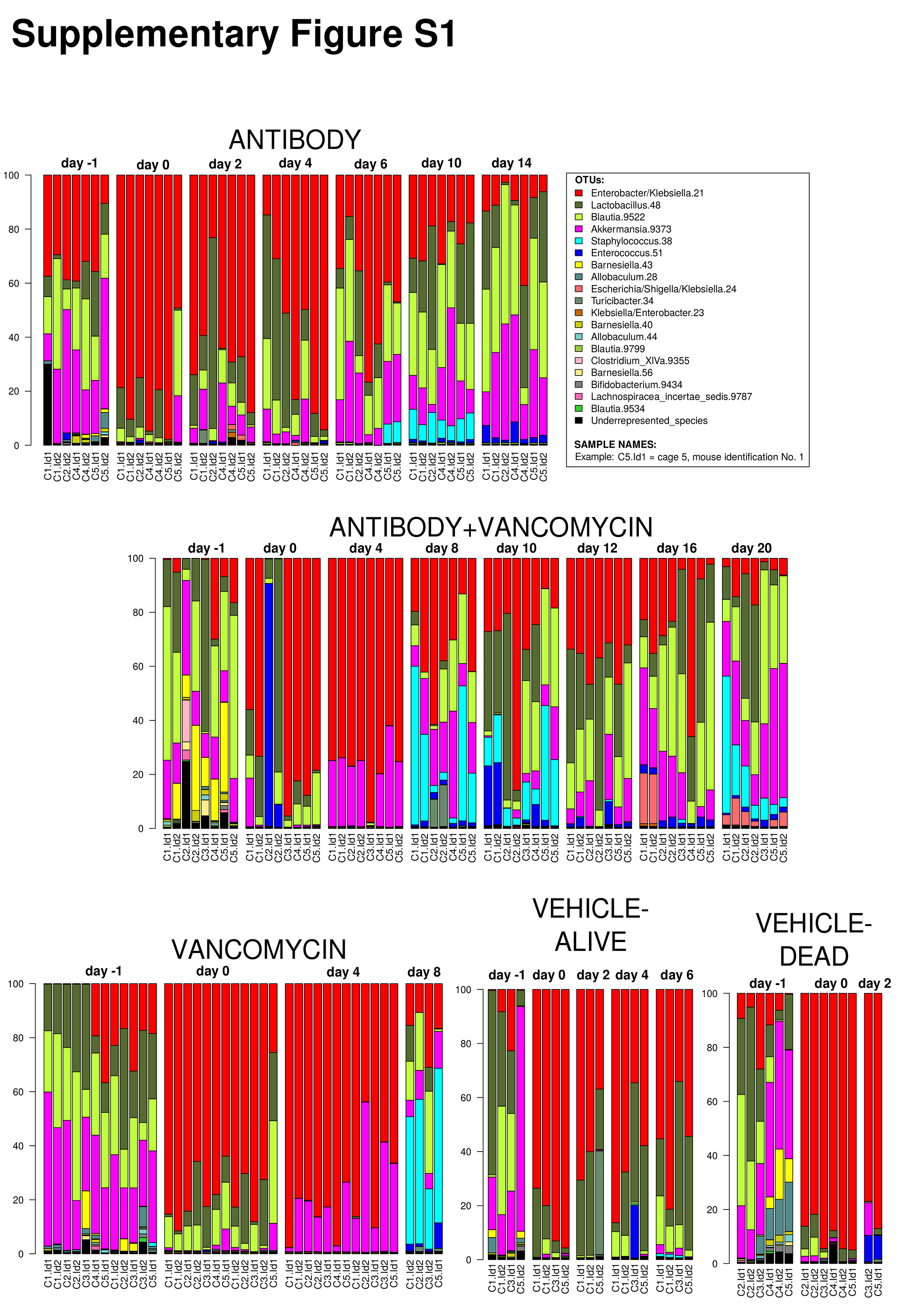

Supplement: Figure S1 — Bacterial proportions of all sequenced samples. Bar-plots showing the percentage proportion of the 19 most prevalent bacterial OTUs. The OTUs summing <7% in total of all 183 samples together are joined into “Underrepresented_species.” [file Image1.TIFF]
